# Alcohol Use Among Young Adults in Northern California During the COVID-19 Pandemic—An Electronic Health Records-Based Study

**DOI:** 10.3389/fpsyt.2022.883306

**Published:** 2022-07-12

**Authors:** Verena E. Metz, Vanessa A. Palzes, Felicia W. Chi, Cynthia I. Campbell, Stacy A. Sterling

**Affiliations:** ^1^Center for Addiction and Mental Health Research, Kaiser Permanente Division of Research, Oakland, CA, United States; ^2^Department of Psychiatry and Behavioral Sciences, University of California, San Francisco, San Francisco, CA, United States; ^3^Department of Health Systems Science, Kaiser Permanente Bernard J. Tyson School of Medicine, Pasadena, CA, United States

**Keywords:** young adults, alcohol use, COVID-19 pandemic, screening, primary care

## Abstract

**Background:**

Individuals globally were affected by the COVID-19 pandemic in myriad of ways, including social isolation and economic hardship, resulting in negative impacts on mental health and substance use. Young adults have been subjected to extraordinary challenges such as job loss, virtual school, or childcare issues, but have received limited attention from research so far.

**Methods:**

Using electronic health record data from a large integrated healthcare system in Northern California, this longitudinal observational study examined changes in the prevalence of unhealthy alcohol use (identified *via* systematic alcohol screening in adult primary care) from pre- (3/1/2019–12/31/2019) to post-COVID onset (3/1/2020–12/31/2020) among young adults (18–34 years). Among the 663,111 and 627,095 young adults who utilized primary care in the pre- and post-COVID onset periods, 342,889 (51.9%) and 186,711 (29.8%) received alcohol screening, respectively. We fit generalized estimating equation Poisson models to estimate the change in prevalence of unhealthy alcohol use from pre- to post-COVID onset among those who were screened, while using inverse probability weighting to account for potential selection bias of receiving alcohol screening. Heterogeneity in the change of prevalence by patient characteristics was also examined.

**Results:**

Overall, the unadjusted prevalence of unhealthy alcohol use slightly decreased from 9.2% pre-COVID to 9.0% post-COVID onset. After adjusting for patient covariates, the prevalence of unhealthy alcohol use decreased by about 2% [adjusted prevalence ratio (aPR) = 0.98, 95% CI = 0.96, 1.00]. The prevalence of unhealthy alcohol use increased among women by 8% (aPR = 1.08, 95% CI = 1.06, 1.11), patients 18–20 years by 7% (aPR = 1.07, 95% CI = 1.00, 1.15), and Latino/Hispanic patients by 7% (aPR = 1.07, 95% CI = 1.03, 1.11). While the prevalence of unhealthy alcohol use decreased among men by 12% (aPR = 0.88, 95% CI = 0.86, 0.90), patients 21–34 years by 2% (aPR = 0.98, 95% CI = 0.96, 0.99), White patients by 3% (95% CI = 0.95, 1.00), and patients living in neighborhoods with the lowest deprivation indices by 9% (aPR = 0.91, 95% CI = 0.88, 0.94), their unadjusted prevalence remained higher than their counterparts post-COVID onset. There was no variation in the change of prevalence by comorbid mental health conditions or drug use disorders.

**Conclusions:**

While changes in unhealthy alcohol use prevalence among young adults were small, findings raise concerns over increased drinking among women, those younger than the U.S. legal drinking age, and Latino/Hispanic patients.

## Introduction

The COVID-19 pandemic and its associated preventive public health and containment measures have impacted individuals globally in myriad ways, ranging from job and financial losses, everyday life restrictions through lockdowns, curfews or remote work or school, to disease-related impairments such as illness or death. Life changed drastically within a short period of time, putting a strain on mental health and wellbeing as reports from many countries around the world confirmed (1–5). Increased prevalence of depression, anxiety, stress and insomnia have been reported (6–8), as well as changes in alcohol and other drug use (9). Moreover, individuals have been affected by the pandemic inequitably (2, 10, 11).

Certain subgroups of the population appear to be at higher risk of substance use and mental health issues during the pandemic, particularly children and adolescents (12), younger adults, people who were unemployed (10) or living alone (7), people with poor physical health or pre-existing mental health conditions, and women (13, 14). Specifically, increased alcohol use has been reported among younger and older adults, and among those with higher pre-pandemic alcohol use or higher levels of stress; other studies have found varying results in regard to socio-demographic characteristics (9, 13, 15), possibly due to a lack of broad data collection on a large scale and differences in the populations studied (e.g., older adults, college students, or patients with pre-existing mental health conditions). This lack of comprehensive data makes targeted prevention, screening, identification, and early intervention efforts for (subgroups of) the general primary care population more challenging.

Alcohol use problems can be effectively identified in primary care (16, 17) with adequate screening, as many people, particularly in high and middle-income countries have access to regular primary care; however, there are still significant gaps in the literature about how alcohol use, other mental health issues, and health care utilization have changed since pandemic onset. Thus, more research is needed for healthcare systems to prepare for adequate screening and intervention efforts, particularly for young adults. Importantly, patterns of alcohol use are frequently established during young adulthood, and thus, there are opportunities to intervene and change people's trajectories of drinking during this period of vulnerability (18). Moreover, young adults may have been very severely affected by the pandemic due to factors such as less job security, housing stability, and higher debt compared to other age groups. Previous studies have reported higher distress and mental health issues caused by the pandemic and its containment measures in younger adults (19–21). Therefore, the aim of this study was to examine impacts of the COVID-19 pandemic on alcohol use problems among a diverse, large sample of young adults (18–34 years) who utilized primary care. Specifically, we compared changes in prevalence of unhealthy alcohol use from pre- (March 1, 2019 to December 31, 2019) to post-COVID onset (March 1, 2020 to December 31, 2020), and potential heterogeneity by patient-level factors, including sex, age group (18–20 vs. 21–34), race/ethnicity, socio-economic status, as well as mental health conditions and healthcare utilization data.

## Methods

### Setting

Kaiser Permanente Northern California (KPNC) is a large, integrated health care delivery system that serves approximately one-third of the Northern Californian population (>4 million members). The membership is racially and ethnically diverse and reflects the U.S. population with access to care (22). KPNC provides care to a population insured through employer-based plans, Medicare, Medicaid, and health insurance exchanges. KPNC has 21 medical centers, 233 medical offices, and 2,147 adult primary care physicians and providers, and provides specialty psychiatry and addiction treatment as a covered benefit (23).

KPNC has had systematic annual alcohol screening in adult primary care since June 2013. Medical assistants ask patients about their alcohol use, including a modified version of the evidence-based National Institute on Alcohol Abuse and Alcoholism (NIAAA) single-item screening question (tailored to the patient's age and sex) to determine the number of heavy drinking days in the past 3 months (i.e., 4 or more drinks/day for women aged ≥18 years and men ≥66 years, or 5 or more drinks/day for men 18–65 years), and two questions to calculate average weekly alcohol consumption (i.e., average number of drinking days per week, and typical number of drinks per drinking day). Prior to the COVID-19 pandemic, average alcohol screening rates were 95%, which decreased to 61% in the second quarter (April to June) of 2020, but then increased up to 70% for the remainder of the year. Importantly, the primary setting for alcohol screening changed from in-person to video visits post-COVID onset as patients began utilizing primary care virtually due to COVID-19 mitigation efforts.

The KPNC Institutional Review Board reviewed the study and granted a waiver of informed consent to examine electronic health record (EHR) data.

### Study Period and Sample

We examined two time periods: pre-COVID (March 1, 2019 to December 31, 2019) and post-COVID onset (March 1, 2020 to December 31, 2020). For each time period, we identified a cohort of young adult KPNC members who were age 18–34 years at the beginning of the period and had at least one primary care visit during the period (pre-COVID *n* = 696,185; post-COVID onset *n* = 643,856). For each cohort, we excluded patients who had <50% membership coverage during the respective period (*n* = 33,048 and *n* = 16,723 for pre- and post-COVID onset, respectively) and patients with unknown sex (*n* = 26 and *n* = 38, respectively), resulting in an analytical sample of 663,111 young adults pre-COVID and 627,095 young adults post-COVID onset.

### Measures

The outcome measure was unhealthy alcohol use, which was identified at any alcohol screening during each time period and defined as exceeding either the daily (≥5 drinks/day for men, or ≥4 drinks/day for women) or weekly drinking limit (>14 drinks/week for men, or >7 drinks/day for women) based on the NIAAA drinking guidelines (24). Patient demographic characteristics (age at the beginning of the period, sex, and race/ethnicity) were extracted from the EHR. We utilized the neighborhood deprivation index (NDI) as a proxy measure of socioeconomic status, which includes indicators of education, income and poverty, employment, housing, and occupation from the 2019 Census' American Community Survey for census tracts (25). Patients' first recorded residential address during their respective cohort period were geocoded to census tracts to obtain their NDI. We categorized NDI into quartiles of each cohort's distribution. We identified common medical conditions, mental health conditions, and drug use disorders based on *International Classification of Diseases and Related Health Problems, 10th Edition* (ICD-10) codes documented at encounters with the health system during each patient's respective cohort period. We created a count variable for the number of medical conditions common among primary care patients, which included asthma, atherosclerosis, atrial fibrillation, chronic kidney disease, chronic liver disease, chronic obstructive pulmonary disease, coronary disease, diabetes, dementia, epilepsy, gastroesophageal reflux, heart failure, hyperlipidemia, hypertension, migraine, osteoarthritis, osteoporosis, osteopenia, Parkinson's disease/syndrome, peptic ulcer, and rheumatoid arthritis) (26, 27). We created binary indicators for any mental health condition (including depression, bipolar disorder, anxiety disorder, obsessive compulsive disorder, post-traumatic stress disorder, schizophrenia, schizoaffective disorder, and attention-deficit/hyperactivity disorder) and any drug use disorder (for cannabis, cocaine, hallucinogens, inhalants, multi-drugs, opioids, sedative/hypnotic/anxiolytics, stimulants, and other drugs).

### Inverse Probability Weighting for Alcohol Screening

Among the 663,111 and 627,095 young adults who utilized primary care in the pre- and post-COVID onset periods, 342,889 (51.9%) and 186,711 (29.8%) received alcohol screening, respectively. We examined potential selection bias due to lack of screening (resulting in missing outcome data) by calculating standardized mean differences (SMDs) in patient characteristics between those who did and did not receive alcohol screening in each cohort. We found meaningful differences in the distributions of sex and medical comorbidities between those who did and did not receive screening (SMDs>0.2) (28, 29). Specifically, the proportion of women among all screened patients was smaller than that among unscreened patients in the pre-COVID cohort (54.3 vs. 67.1%, SMD = −0.264) and the proportion of patients with no medical comorbidities among all screened patients was smaller than that among unscreened patients in the post-COVID onset cohort (68.4 vs. 78.7%, SMD = −0.235; Supplementary Table 1).

To account for the potential selection bias, we estimated and applied inverse probability weights (IPWs) for receiving alcohol screening for each patient in each cohort. The IPWs were based on the predicted probabilities of being screened (separately for each cohort), estimated with logistic regression models, conditional on patient characteristics (age, sex, race/ethnicity, type of insurance, NDI quartile, number of medical conditions, any mental health condition, and any drug use disorder). The weights for screened patients in both cohorts had reasonable distributions (pre-COVID: mean = 1.9, median = 1.9, min = 1.0, max = 3.3; post-COVID onset: mean = 3.4, median = 3.4, min = 1.0, max = 6.3) and were successful at balancing patient characteristic distributions between those who did and did not receive alcohol screening (all absolute SMD < 0.2; Supplementary Table 1). The sum of the weights was 662,517 patients pre-COVID and 625,840 patients post-COVID.

### Statistical Analysis

Analyses were conducted in three steps. Firstly, we estimated overall weighted unadjusted prevalence of unhealthy alcohol use during pre-COVID (March 1, 2019 to December 31, 2019) and post-COVID onset (March 1, 2020 to December 31, 2020) periods with an IPW-weighted Poisson generalized estimating equation (GEE) model. Poisson regression allowed us to estimate prevalence of more common binary outcomes rather than odds (30–32), and we utilized GEE with robust standard error estimation to adjust for the within-patient correlation of repeated measures [there were 405,547 (45%) patients in both cohorts] while accounting for lack of independence in replications of patients induced by weighting (33). Secondly, we estimated the change in prevalence of unhealthy alcohol use from pre- to post-COVID onset (i.e., prevalence ratio) by refitting the weighted Poisson GEE model, adjusting for sex, age group (18–20 vs. 21–34), race/ethnicity, type of insurance, NDI quartile, number of medical conditions, any mental health condition, and any drug use disorder. A very small proportion of patients had unknown NDI (0.2%), which we imputed as the cohort's mean and assigned to the third quartile.

Thirdly, to examine potential heterogeneity in the change of unhealthy alcohol use prevalence from pre- to post-COVID onset by patient characteristics (sex, age group, race/ethnicity, NDI quartile, mental health conditions, and drug use disorders), we included an interaction term between the indicator variable for time (post-COVID = 1) and the covariate, individually in separate models. For each of the patient characteristics, we first fit an IPW-weighted Poisson GEE models without additional covariates to estimate unadjusted prevalence of unhealthy alcohol use during pre-COVID and post-COVID onset periods for the patient subgroups, then refit the model by including all other patient covariates to estimate adjusted prevalence ratios for the patient subgroups and evaluated whether the Type 3 test for the interaction terms had *p* < 0.05. All statistical analyses were conducted using SAS software, Version 9.4 (SAS Institute Inc., Cary, NC).

## Results

### Cohort Characteristics

The pre-COVID cohort of young adult (18–34 years) KPNC members with primary care utilization (*n* = 663,111) was 60.4% female, 86.4% age 21–34 years, 37.2% White, 23.3% Asian/Native Hawaiian/Pacific Islander (API), 8% African American/Black, 25.8% Latino/Hispanic, 0.8% Native American, and 4.8% other/unknown race/ethnicity (Table 1). The majority of the pre-COVID cohort had commercial insurance (86.5%). About 18.6% of the pre-COVID cohort had one medical condition and 7.2% had two or more. The prevalence of drug use disorders (DUDs) was 1.4% and mental health conditions was 19.2%. The post-COVID cohort of young adult KPNC members with primary care utilization (*n* = 627,095) had similar distributions of patient demographic and clinical characteristics as the pre-COVID cohort.

**Table 1:** Characteristics of young adult (18–34 years) KPNC members who utilized primary care pre-COVID (March to December 2019) and post-COVID onset (March to December 2020).

**Characteristic**	**Pre-COVID** **(*n* = 663,111)**	**Post-COVID onset** **(*n* = 627,095)**
Sex, *n* (%)		
Male	262,291 (39.6)	252,260 (40.2)
Female	400,820 (60.4)	374,835 (59.8)
Age (years), mean (SD)	26.8 (4.8)	26.8 (4.8)
Age group (years), *n* (%)		
18–20	90,453 (13.6)	85,335 (13.6)
21–34	572,658 (86.4)	541,760 (86.4)
Race/ethnicity, *n* (%)		
White	246,709 (37.2)	224,708 (35.8)
API	154,569 (23.3)	133,712 (21.3)
Black	53,185 (8.0)	51,376 (8.2)
Latino/Hispanic	171,126 (25.8)	171,029 (27.3)
Native American	5,542 (0.8)	5,252 (0.8)
Other/Unknown	31,980 (4.8)	41,018 (6.5)
Type of insurance, *n* (%)		
Medicaid	48,562 (7.3)	46,999 (7.5)
Medicare	4,141 (0.6)	4,235 (0.7)
Commercial	573,478 (86.5)	562,788 (89.7)
Other/Unknown	36,930 (5.6)	13,073 (2.1)
Neighborhood deprivation index		
quartile, *n* (%)		
Q1 (lowest)	170,164 (25.7)	154,601 (24.7)
Q2	169,171 (25.5)	158,183 (25.2)
Q3	165,123 (24.9)	156,686 (25.0)
Q4 (highest)	157,413 (23.7)	156,417 (24.9)
Unknown	1,240 (0.2)	1,208 (0.2)
Number of medical conditions, *n* (%)		
0	491,444 (74.1)	474,137 (75.6)
1	123,608 (18.6)	110,920 (17.7)
≥2	48,059 (7.2)	42,038 (6.7)
Drug use disorder, *n* (%)^a^	9,609 (1.4)	9,702 (1.5)
Mental health condition, *n* (%)^a^	127,261 (19.2)	126,005 (20.1)
Had an alcohol screening, *n* (%)	343,889 (51.9)	186,711 (29.8)

*API, Asian, Native Hawaiian, or Pacific Islander; KPNC, Kaiser Permanente Northern California.*

a *Diagnoses were identified via ICD-10 diagnoses codes during each respective time period*.

### Changes in Prevalence of Unhealthy Alcohol Use

Among the IPW-weighted samples of young adults, the overall prevalence of unhealthy alcohol use slightly decreased from 9.2% (95% CI = 9.1, 9.3) pre-COVID to 9.0% (95% CI = 8.9, 9.2) post-COVID onset (Table 2). After adjusting for patient characteristics, this change represented a slight, but significant decrease of about 2% [adjusted prevalence ratio (aPR) = 0.98, 95% CI = 0.96, 1.00]. There was variation in the change of prevalence by several patient characteristics, including sex, age group, race/ethnicity, and NDI quartile, but not by comorbid mental health conditions or drug use disorders. Specifically, the prevalence of unhealthy alcohol use increased among women by 8% (aPR = 1.08, 95% CI = 1.06, 1.11), patients 18–20 years by 7% (aPR = 1.07, 95% CI = 1.00, 1.15), and Latino/Hispanic patients by 7% (aPR = 1.07, 95% CI = 1.03, 1.11). In contrast, the prevalence of unhealthy alcohol use decreased among men by 12% (aPR = 0.88, 95% CI = 0.86, 0.90), patients 21–34 years by 2% (aPR = 0.98, 95% CI = 0.96, 0.99), and patients living in neighborhoods with the lowest NDIs by 9% (aPR = 0.91, 95% CI = 0.88, 0.94). There were also decreases in prevalence of unhealthy alcohol use among White, API, Black, and Native American patients, with the greatest decrease among Native American patients (aPR = 0.78, 95% CI = 0.63, 0.97). There were no significant changes in prevalence among patients with other/unknown race/ethnicity and patients living in neighborhoods with higher NDIs. Despite differential changes in prevalence by these characteristics, IPW-weighted unadjusted prevalence of unhealthy alcohol use post-COVID remained higher among men compared to women (10.3 vs. 8.2%), patients aged 21–34 years compared to patients aged 18–20 years (9.7 vs. 4.9%), White patients compared to other racial/ethnic groups (11.6 vs. 5.8–8.9%), and patients living in neighborhoods with the lowest NDIs compared to those living in neighborhoods with higher NDIs (9.9 vs. 8.5–9.2%).

**Table 2:** Unadjusted prevalence and adjusted prevalence ratios (aPR) of unhealthy alcohol use, overall and by patient characteristics, among young adult KPNC members who were screened, weighted by IPW.

**Group**	**Pre-COVID (*n* = 662,517),** **% (95% CI)^**a**^**	**Post-COVID onset (*n* = 625,840),** **% (95% CI)^**a**^**	**Post vs. Pre, aPR (95% CI)^**b**^**	**Type 3** ***p*-value^**c**^**	***p*-value**
Overall	9.2 (9.1, 9.3)	9.0 (8.9, 9.2)	0.98 (0.96, 1.00)	-	0.043
By sex				<0.001	
Male	11.6 (11.5, 11.8)	10.3 (10.1, 10.5)	0.88 (0.86, 0.90)		<0.001
Female	7.5 (7.4, 7.7)	8.2 (8.0, 8.3)	1.08 (1.06, 1.11)		<0.001
By age group (years)				0.007	
18–20	4.5 (4.3, 4.7)	4.9 (4.6, 5.1)	1.07 (1.00, 1.15)		0.037
21–34	9.9 (9.8, 10.0)	9.7 (9.5, 9.8)	0.98 (0.96, 0.99)		0.007
By race/ethnicity				<0.001	
White	11.9 (11.8, 12.1)	11.6 (11.4, 11.9)	0.97 (0.95, 1.00)		0.047
API	6.4 (6.2, 6.5)	5.8 (5.6, 6.1)	0.91 (0.87, 0.96)		<0.001
Black	8.0 (7.7, 8.4)	7.4 (7.0, 7.9)	0.92 (0.86, 0.99)		0.021
Latino/Hispanic	8.1 (7.9, 8.3)	8.7 (8.5, 9.0)	1.07 (1.03, 1.11)		<0.001
Native American	8.1 (7.2, 9.2)	6.4 (5.4, 7.8)	0.78 (0.63, 0.97)		0.028
Other/Unknown	9.2 (8.7, 9.6)	8.9 (8.4, 9.5)	0.97 (0.89, 1.04)		0.380
By NDI quartile				<0.001	
Q1 (lowest)	10.9 (10.7, 11.1)	9.9 (9.6, 10.2)	0.91 (0.88, 0.94)		<0.001
Q2	9.2 (9.0, 9.4)	9.2 (8.9, 9.4)	0.99 (0.96, 1.03)		0.702
Q3	8.4 (8.3, 8.6)	8.7 (8.4, 8.9)	1.02 (0.98, 1.06)		0.368
Q4 (highest)	8.1 (7.9, 8.2)	8.5 (8.2, 8.7)	1.04 (1.00, 1.08)		0.071
By mental health condition				0.898	
None	8.9 (8.8, 9.0)	8.8 (8.7, 9.0)	0.98 (0.96, 1.00)		0.072
Any	10.2 (10.0, 10.4)	10.0 (9.7, 10.3)	0.98 (0.95, 1.02)		0.357
By drug use disorder				0.120	
None	9.1 (9.0, 9.2)	9.0 (8.9, 9.1)	0.98 (0.97, 1.00)		0.075
Any	13.7 (12.8, 14.7)	12.6 (11.5, 13.8)	0.90 (0.80, 1.01)		0.067

*API, Asian, Native Hawaiian, or Pacific Islander; KPNC, Kaiser Permanente Northern California; IPW, Inverse Probability Weights.*

a *The sample size is the sum of the weights of the IPW procedure.*

b *Models were adjusted for sex, age group, race/ethnicity, type of insurance, NDI quartile, number of medical conditions, any mental health condition, and any drug use disorder.*

c *Type 3 p-value for the corresponding interaction term(s) between the patient characteristic and the pre/post indicator*.

## Discussion

Among a large sample of young adults in Northern California who utilized primary care, we found a small, but significant, overall decrease in prevalence of unhealthy alcohol use following onset of the COVID-19 pandemic compared to the year prior; however, this decrease was not equal across patient subgroups, underscoring the importance of examining heterogeneity in alcohol use studies. The overall decrease in prevalence appears to have been largely driven by men; patients aged 21–34 years, White, API, Black, and Native American patients; and patients living in neighborhoods with the lowest NDIs (higher SES); some of these subgroups (men, White, Black, and Native American patients) have typically had higher prevalence pre-COVID (34). In contrast, the prevalence of unhealthy alcohol use increased slightly, with similar magnitude, among young women, those aged 18–20 years (below the legal drinking age in the U.S.) and Latino/Hispanic young adults, pre- to post-COVID onset. These data cannot tell us why unhealthy alcohol increased among subgroups, but stress, anxiety, depression, social isolation, and suicidal thoughts elicited by the pandemic might have played a role, since these factors have been associated with changes in the consumption of alcohol and other substances (9, 35–37). Some of these factors may be more prominent in certain subgroups. For example, studies have found that level of alcohol problems was associated with loneliness only for women (38), and significant associations between early-COVID-19 period, depression and anxiety symptoms, and drinking to forget one's worries among women only (39). Research also found higher psychological distress among young adults aged 18–24 years and Hispanic/Latino individuals during the pandemic (40, 41). Moreover, the national Monitoring the Future study (42) that examined historical trends and potential 2020 shifts in prevalence and frequency of drinking, also reflected the downward trend in past 30-day alcohol use in young adults that we found in California; however, the authors also noted more binge drinking compared to prior to the pandemic as well as changes in drinking contexts and reasons among drinkers (such as increased boredom, drinking to relax, and drinking while alone).

While effect sizes for these changes in prevalence of unhealthy alcohol use were very small, the increases observed among young adult women, adults younger than the U.S. legal drinking age, and Latino/Hispanic young adults in our study reflect national trends (43–48), which are concerning for several reasons. Whereas, Latino/Hispanic members in the U.S. are generally less likely to drink compared to non-Hispanic Whites, those who drink tend to drink larger quantities (49). This dynamic is even more apparent in subgroups with higher levels of acculturation to the American culture, those who have more relaxed attitudes toward drinking and young women with Latino/Hispanic background (49). Moreover, members of the Latino/Hispanic communities tend to be less likely to seek treatment for alcohol-related problems (49). In general, women are more likely than men to progress faster to AUDs and experience more serious alcohol-related health problems (50, 51). Several recent studies have shown that women in the U.S. are increasingly drinking more and that the gender gap is narrowing, particularly among the younger cohorts (34, 52, 53). Thus, our pandemic-related findings may indeed reflect a troubling closing in the historical gaps in drinking behaviors between socio-demographic subgroups, and thus, an overall trend in our society (54).

Our findings provide important information for health systems and could help inform targeted screening and brief intervention efforts. The finding that unhealthy alcohol use may be increasing among vulnerable sub-groups even in the context of overall (54) decreases suggests that clinicians may need to focus increased attention on groups which may not have been perceived at high risk of unhealthy alcohol use in the past. Primary care providers of SBIRT (screening, brief intervention, referral to treatment) may use this information to assess the potential need in their young adult patients for more targeted (early) interventions or prevention programs. Particularly now, as telemedicine has become more common during the pandemic, there may be more opportunities to provide SBIRT through telehealth/virtual approaches, which may appeal to these vulnerable young adults and increase ways for clinicians to reach young people and help them develop healthier behavioral alternatives. More research examining utilization and effectiveness of telehealth services among young adults following onset of the COVID-19 pandemic is greatly needed.

Our study has a number of limitations. Alcohol use was self-reported and thus subject to potential under-reporting. Additionally, alcohol use could only be assessed among members who had primary care visits and were screened during the study periods. Patients may not have been screened during the study period if they were not due for screening (screening is conducted annually, or every 6 months if a prior screening indicates unhealthy alcohol use) or other reasons such as busy workflows or medical assistants forgetting to administer the screening instrument. In addition, the percentage of young adults screened in primary care was lower post-pandemic onset compared to the year prior; this is most likely due to the pandemic (onset) interrupting clinical workflows, and the need to adapt and shift to virtual care. Although there were some differences between those who were screened vs. not screened, we adjusted for potential selection bias with a propensity score weighting approach that is appropriate for re-balancing characteristics of the study sample. However, generalizability of the study findings remains limited to young adults who use primary care services. In addition, there may be moderating factors that we are not able to measure with our data such as acute individual stressors or social determinants of mental health and substance use (e.g., housing or job insecurity or childcare issues). We did not find any significant associations between mental health and drug use and unhealthy alcohol use prevalence changes in our sample, suggesting that other factors might play a role in this regard. The reasons why certain subgroups had differential changes in prevalence of unhealthy alcohol use pre- to post-pandemic onset are critical for future studies to examine.

Our analysis of changes in self-reported drinking behavior in this large, socio-demographically diverse sample of young adults revealed important trends in regard to unhealthy alcohol use. Groups who may have engaged in less unhealthy use in the past, e.g., young women compared to their male counterparts, may be closing these gaps and putting themselves at increased risk of alcohol-related harms as they have higher prevalence of unhealthy alcohol use post-COVID onset. These vulnerable groups might need special attention or targeted efforts to prevent further progression of this trend as the pandemic continues to impact our everyday lives.

## Data Availability Statement

The datasets presented in this article are not readily available because the analytical datasets from this project include patient level data from the KPNC EHR, administrative and clinical databases. External investigators can contact the study PI to initiate a request for study data to support new study proposals or article. Approval of such requests and initiation of collaborations will consider the following criteria: The proposed project must be of high scientific merit. The proposed project must be consistent with the overall goals and objectives of the parent study. The proposed project must meet certain participant burden criteria (for any new primary data collection involving subjects), including: Acceptable to the subjects (e.g., risks, time, discomfort, and privacy); and not hinder or disrupt clinical care provided by study sites. The proposed project's investigators must plan for adequate resources to effectively complete the project, including: Sufficient budget to cover costs of personnel and supplies; and staff possessing the requisite expertise to meet the objectives of the project. The proposed project should document any involvement of parent study investigators as part of the research team. Approved requests for data will take into account data sharing agreements between KPNC and NIH. Data will be de-identified prior to release for sharing. Requests to access the datasets should be directed to stacy.a.sterling@kp.org.

## Ethics Statement

The studies involving human participants were reviewed and approved by IRB of Kaiser Permanente Northern California. Written informed consent for participation was not required for this study in accordance with the national legislation and the institutional requirements.

## Author Contributions

VM, VP, FC, CC, and SS contributed to the concept and methodology of the study. VP and FC conducted the analysis and were responsible for gathering the relevant data from the electronic health records. CC and SS supervised all publication-related activities and obtained funding for the project. VM conducted the submission. All authors contributed to writing and editing the manuscript and approved the submitted version. All authors agree to be accountable for the content of the work.

## Funding

This research was supported by a grant from the National Institute on Alcohol Abuse and Alcoholism: R01AA027477 (PI: SS).

## Conflict of Interest

The authors declare that the research was conducted in the absence of any commercial or financial relationships that could be construed as a potential conflict of interest.

## Publisher's Note

All claims expressed in this article are solely those of the authors and do not necessarily represent those of their affiliated organizations, or those of the publisher, the editors and the reviewers. Any product that may be evaluated in this article, or claim that may be made by its manufacturer, is not guaranteed or endorsed by the publisher.
