# Supplementary material for: Alcohol Use Among Young Adults in Northern California During the COVID-19 Pandemic—An Electronic Health Records-Based Study
Source: Front Psychiatry. 2022 Jul 12;13:883306. doi: 10.3389/fpsyt.2022.883306 (PMC9315391; doi:10.3389/fpsyt.2022.883306)
Supplement: Supplementary file 1 [file Table_1.DOCX]

| **Supplementary Table 1**. Characteristics of young adult (18-34 years) KPNC members who had a primary care visit, by whether they had an alcohol screening. | | | | | | | | | |
| --- | --- | --- | --- | --- | --- | --- | --- | --- | --- |
|  | **Pre-COVID** | | | | **Post-COVID Onset** | | | |  |
| **Characteristic** | **Screened (n=343,889)** | **Not Screened (n=319,222)** | **SMD before IPW^a^** | **SMD after IPW^a^** | **Screened (n=186,711)** | **Not Screened (n=440,384)** | **SMD before IPW^a^** | **SMD after IPW^a^** |  |
| Sex, n (%) |  |  |  |  |  |  |  |  |  |
| Male | 157,208 (45.7) | 105,083 (32.9) | 0.264 | -0.002 | 80,078 (42.9) | 172,182 (39.1) | 0.077 | 0 |  |
| Female | 186,681 (54.3) | 214,139 (67.1) | -0.264 | 0.002 | 106,633 (57.1) | 268,202 (60.9) | -0.077 | 0 |  |
| Age, mean (SD) | 26.7 (4.8) | 26.9 (4.8) | -0.042 | -0.026 | 26.7 (4.8) | 26.8 (4.8) | -0.017 | -0.019 |  |
| Age group, n (%) |  |  |  |  |  |  |  |  |  |
| 18-20 | 49,122 (14.3) | 41,331 (12.9) | 0.041 | 0 | 26,290 (14.1) | 59,045 (13.4) | 0.02 | 0.003 |  |
| 21-34 | 294,767 (85.7) | 277,891 (87.1) | -0.041 | 0 | 160,421 (85.9) | 381,339 (86.6) | -0.02 | -0.003 |  |
| Race/ethnicity, n (%) |  |  |  |  |  |  |  |  |  |
| White | 126,158 (36.7) | 120,551 (37.8) | -0.023 | 0 | 66,485 (35.6) | 158,223 (35.9) | -0.006 | 0.002 |  |
| API | 82,918 (24.1) | 71,651 (22.4) | 0.040 | 0 | 42,058 (22.5) | 91,654 (20.8) | 0.041 | 0 |  |
| Black | 25,958 (7.5) | 27,227 (8.5) | -0.037 | 0 | 15,265 (8.2) | 36,111 (8.2) | 0 | 0.004 |  |
| Latino/Hispanic | 89,991 (26.2) | 81,135 (25.4) | 0.018 | 0 | 51,484 (27.6) | 119,545 (27.1) | 0.011 | 0 |  |
| Native American | 2,900 (0.8) | 2,642 (0.8) | 0 | 0 | 1,652 (0.9) | 3,600 (0.8) | 0.011 | 0 |  |
| Other/Unknown | 15,964 (4.6) | 16,016 (5.0) | -0.019 | 0 | 9,767 (5.2) | 31,251 (7.1) | -0.079 | 0 |  |
| Type of insurance, n (%) |  |  |  |  |  |  |  |  |  |
| Medicaid | 23,879 (6.9) | 24,683 (7.7) | -0.031 | 0 | 13,852 (7.4) | 33,147 (7.5) | -0.004 | 0 |  |
| Medicare | 2,108 (0.6) | 2,033 (0.6) | 0 | 0 | 1,420 (0.8) | 2,815 (0.6) | 0.024 | 0 |  |
| Commercial | 292,448 (85.0) | 281,030 (88.0) | -0.088 | -0.003 | 167,119 (89.5) | 395,669 (89.8) | -0.01 | 0 |  |
| Other/Unknown | 25,454 (7.4) | 11,476 (3.6) | 0.167 | 0.004 | 4,320 (2.3) | 8,753 (2.0) | 0.021 | 0 |  |
| Neighborhood deprivation index quartile, n (%) |  |  |  |  |  |  |  |  |  |
| Q1 (lowest) | 90,670 (26.4) | 79,494 (24.9) | 0.034 | 0 | 51,131 (27.4) | 103,470 (23.5) | 0.09 | 0 |  |
| Q2 | 87,189 (25.4) | 81,982 (25.7) | -0.007 | 0 | 46,654 (25.0) | 111,529 (25.3) | -0.007 | 0.002 |  |
| Q3 | 84,890 (24.7) | 80,233 (25.1) | -0.009 | 0 | 45,491 (24.4) | 111,195 (25.2) | -0.019 | 0 |  |
| Q4 (highest) | 80,550 (23.4) | 76,863 (24.1) | -0.016 | 0 | 43,105 (23.1) | 113,312 (25.7) | -0.061 | 0 |  |
| Unknown | 590 (0.2) | 650 (0.2) | 0 | 0 | 330 (0.2) | 878 (0.2) | 0 | 0 |  |
| Number of medical conditions, n (%) |  |  |  |  |  |  |  |  |  |
| 0 | 241,168 (70.1) | 250,276 (78.4) | -0.191 | -0.039 | 127,747 (68.4) | 346,390 (78.7) | -0.235 | -0.039 |  |
| 1 | 73,193 (21.3) | 50,415 (15.8) | 0.142 | 0.070 | 40,542 (21.7) | 70,378 (16.0) | 0.146 | 0.049 |  |
| ≥2 | 29,528 (8.6) | 18,531 (5.8) | 0.108 | -0.038 | 18,422 (9.9) | 23,616 (5.4) | 0.170 | -0.016 |  |
| Drug use disorder, n (%) | 5,097 (1.5) | 4,512 (1.4) | 0.008 | 0 | 3,384 (1.8) | 6,318 (1.4) | 0.032 | 0 |  |
| Mental health condition, n (%) | 71,924 (20.9) | 55,337 (17.3) | 0.092 | -0.005 | 46,919 (25.1) | 79,086 (18.0) | 0.173 | 0.002 |  |
| API: Asian, Native Hawaiian, or Pacific Islander.  ^a^ Standardized mean differences (SMD) were calculated to determine whether there were differences between patients who were screened and those who were not screened, before and after inverse probability of screening weights (IPW) were applied. | | | | | | | | | |
